# Performance Analysis With Different Types of Visual Stimuli in a BCI-Based Speller Under an RSVP Paradigm

**DOI:** 10.3389/fncom.2020.587702

**Published:** 2021-01-05

**Authors:** Ricardo Ron-Angevin, M. Teresa Medina-Juliá, Álvaro Fernández-Rodríguez, Francisco Velasco-Álvarez, Jean-Marc Andre, Veronique Lespinet-Najib, Liliana Garcia

**Affiliations:** ^1^UMA-BCI Group, Departamento de Tecnología Electrónica, Universidad de Málaga, Malaga, Spain; ^2^Laboratoire IMS, CNRS UMR5218, Cognitique Team, Bordeaux INP-ENSC, Talence, France

**Keywords:** brain computer-interface (BCI), rapid serial visual presentation (RSVP), electroencephalography (EEG), P300, N170, famous faces, neutral pictures

## Abstract

Brain-Computer Interface (BCI) systems enable an alternative communication channel for severely-motor disabled patients to interact with their environment using no muscular movements. In recent years, the importance of research into non-gaze dependent brain-computer interface paradigms has been increasing, in contrast to the most frequently studied BCI-based speller paradigm (i.e., row-column presentation, RCP). Several visual modifications that have already been validated under the RCP paradigm for communication purposes have not been validated under the most extended non-gaze dependent rapid serial visual presentation (RSVP) paradigm. Thus, in the present study, three different sets of stimuli were assessed under RSVP, with the following communication features: white letters (WL), famous faces (FF), neutral pictures (NP). Eleven healthy subjects participated in this experiment, in which the subjects had to go through a calibration phase, an online phase and, finally, a subjective questionnaire completion phase. The results showed that the FF and NP stimuli promoted better performance in the calibration and online phases, being slightly better in the FF paradigm. Regarding the subjective questionnaires, again both FF and NP were preferred by the participants in contrast to the WL stimuli, but this time the NP stimuli scored slightly higher. These findings suggest that the use of FF and NP for RSVP-based spellers could be beneficial to increase information transfer rate in comparison to the most frequently used letter-based stimuli and could represent a promising communication system for individuals with altered ocular-motor function.

## Introduction

Brain computer interfaces (BCI) was first described by Vidal (1973) as a man-computer dialogue using observable and controllable neuroelectric events. That is, BCIs are a type of system that allow users to interact with their environment, using no muscular movements but only their brain activity (Nicolas-Alonso and Gomez-Gil, 2012). Therefore, these systems serve as a last communication channel between severely motor-disabled patients, such as amyotrophic lateral sclerosis (ALS) patients or those with brainstem injuries in a locked-in state (LIS), and their environment.

The most frequently control signal BCI systems use is the brain bioelectricity recorded through electroencephalography (EEG) (Nicolas-Alonso and Gomez-Gil, 2012; Rezeika et al., 2018). The EEG data is processed, and different brain components could be studied depending on the stimulus type and system that is desired to be controlled. The most typical components used in BCI are steady-state visual evoked potentials (SSVEP), event-related potentials (ERP) and sensorimotor rhythms (SMR) (Nicolas-Alonso and Gomez-Gil, 2012). The present study will focus on ERP components, which are evoked after the appearance of an infrequent stimulus. The most studied component of this type is the P300 component, which was first discovered by Sutton et al. (1965) and described as a positive amplitude waveform alteration that reaches peak amplitude at about 300 ms after a sensory stimulus. This potential is mostly recorded in the parietal area (Polich, 2007).

This P300 component is usually employed as a control signal for a type of BCI system which is called a virtual speller (Rezeika et al., 2018). The first P300 based BCI speller was proposed by Farwell and Donchin (1988). This speller consisted of a 6 × 6 matrix table of letters and numbers, whose rows and columns were highlighted (i.e., the characters color turned from gray to white) pseudorandomly in order to evoke the P300 component each time the target character was highlighted. As a consequence, this BCI speller presentation paradigm is called row-column paradigm (RCP). On the other hand, to consistently elicit and classify the P300 component, users are often asked to focus their attention on their desired target letter and count the number of times it flashes, and a classification algorithm differentiates the target letter between many non-targets. Other temporal components generated earlier or later to P300 (P100, N170, N250, N400) are equally analyzed to detect stimuli features (Zheng et al., 2012; Jiang et al., 2017; Tian et al., 2018).

Different variations on the highlighting type and nature of the characters have been studied, such as the shape, color and size of the characters (Salvaris and Sepulveda, 2009; Ryan et al., 2017; Fernández-Rodríguez et al., 2019b) in order to improve system performance (classification accuracy, information transfer rate or ERP amplitude). Regarding the nature of stimuli, it has been demonstrated that the presentation of famous faces (FF) instead of letters leads to an improvement in performance (Kaufmann et al., 2011; Li et al., 2015). Other set of images, such as neutral images, might also help to increase performance as compared to letters (Fernández-Rodríguez et al., 2019a). Moreover, the study of Kellicut-Jones and Sellers (2018) suggests that the FF paradigm might not be significantly better than neutral images in RCP. On the other hand, in the single character presentation (SCP) paradigm –which consists of illuminating the matrix stimuli one by one– the use of faces (non-famous) seemed to increase performance as compared to neutral images (inanimate objects) (Zhao et al., 2011). Nevertheless, these study results should be carefully considered as they are derived from a small sample size. Even though this study is not completely adequate, these findings might suggest that a difference in performance might exist depending on the stimulus presentation paradigm used, in particular when applying FF and neutral images.

The stimulus presentation paradigms RCP and SCP present their stimuli in different locations of the monitor screen, but RCP presents them by row and column groups, and SCP, individually. However, this type of presentation paradigm might not be the most suitable for some patients with motor disabilities who also have no or residual ocular mobility, as the performance of these paradigms is greatly decreased under covert attention conditions (Brunner et al., 2010; Treder and Blankertz, 2010). Different type of visual gaze-independent BCIs have been researched by the literature in order to prevent this limitation. According to the BCI-Spellers review by Rezeika et al. (2018), two groups of main gaze-independent spellers have been proposed by previous literature: (i) those that display the stimuli to be selected in different close positions to control the speller under covert attention, such as Chroma Speller (Acqualagna et al., 2013), Geospell (Aloise et al., 2012), Gaze-Independent Block Speller (GIBS) (Pires et al., 2011) and Hex-O-Spell (Treder and Blankertz, 2010); or (ii) those based on rapid serial visual presentation (RSVP), which sequentially presents stimuli in the center of the screen (Acqualagna and Blankertz, 2013). The authors of this review stated that the RSVP-based BCIs show promising results and have been the most widely used to date.

Different visual configurations of the stimuli under RSVP had also been studied in the literature to increase the system performance for different applications like face recognition or RSVP spellers (Lees et al., 2018). In a recent study, Chen et al. (2016) tested if the characteristics of the stimuli can affect the performance of the system using colored balls, gray dummy faces and colored dummy faces. For each paradigm, six different stimuli were presented (six colors and six dummy face expressions). They found that the combination of colors and dummy face expressions could improve the bit rate. Regarding RSVP spellers, a previous study found a trend in which using colors and different capitalizations might improve the accuracy and bit rate compared to black letters (Acqualagna and Blankertz, 2013). Furthermore, the study of Won et al. (2018) proposed a RSVP speller whose colored stimuli were placed in six different near central positions. They found that using different locations for the letters increased the accuracy of the system in contrast to the classical RSVP paradigm.

Nevertheless, studies regarding the nature of the stimuli under RSVP have barely been carried out for communication purposes (i.e., RSVP spellers). In a preliminary study, neutral images and letters were compared in RSVP (Fernández-Rodríguez et al., 2019c). The results of this work showed that neutral images did not offer significant benefits as compared to letters under the RSVP paradigm. In the same way, to our knowledge, any studies regarding RSVP have compared FF to letters and it would be interesting to determine the efficacy of FF under a RSVP paradigm. However, the results of Fernández-Rodríguez et al. (2019c) should also be carefully considered as a small sample size was applied and no metrics regarding the user experience were considered (such as fatigue, preference and control). To better understand the effect of this sort of stimuli when applying an RSVP paradigm, an extended and complete study regarding neutral images and FF against letters should be carried out.

We hypothesized that using alternative stimuli under RSVP –i.e., famous faces and neutral pictures– instead of letters would increase system performance and user experience of the RSVP-based spellers, as previously demonstrated in the RCP and SCP presentation paradigms. Therefore, the aim of this study was to compare and evaluate the performance of three different types of stimuli (letters, famous faces and neutral pictures) as feasible communication stimuli for a gaze-independent BCI speller. The evaluation was carried out in terms of objective parameters (specifically, accuracy, information transfer rate and brain waveform analysis) and a subjective questionnaire regarding the perception of the participants. The main contribution of this study would be to experimentally (in)validate the usability of alternative stimuli under the RSVP paradigm for communication purposes.

## Methods

### Participants

Eleven French participants (aged 19.91 ± 0.83) took part in the present study. None of the participants had previous experience in the use of BCI systems. The study was approved by the Ethics Committee of the University of Malaga and met the ethical standards of the Helsinki Declaration. According to self-reports, none of the participants had any history of neurological or psychiatric illness. In addition, all of them provided written consent trough a protocol reviewed by the ENSC-IMS (Ecole Nationale Supérieur de Cognitique – Intégration du Matériau su Système) Cognitive and UMA-BCI teams.

### Data Acquisition and Signal Processing

The EEG was recorded using the electrode positions: Fz, Cz, Pz, Oz, P3, P4, PO7, and PO8, according to the 10/20 international system. All channels were referenced to the right earlobe, using FPz as the ground.

The EEG was amplified through a 16 channel biosignal amplifier gUSBamp (Guger Technologies). The amplifier settings were from 0.5 to 100 Hz for the band-pass filter, the notch (50 Hz) was on, and the sensitivity was 500 μV. The signal was then digitized at a rate of 256 Hz. EEG data collection and processing were controlled by the *UMA-BCI Speller* software (Velasco-Álvarez et al., 2019), which serves as the front-end to BCI2000 (Schalk et al., 2004). Likewise, when the brain signal was recorded by the *UMA-BCI Speller*, a pass-band filter from 0.1 to 60 Hz was applied, and the notch filter was on at 60 Hz.

A stepwise linear discriminant analysis (SWLDA) of the data was performed to obtain the weights for the P300 classifier and calculate the accuracy. Alternative classification methods of the EEG signal have been proposed by the literature (Lotte et al., 2018; Xiao et al., 2020), however the SWLDA algorithm has been widely used and validated (Krusienski et al., 2008; Lees et al., 2018). Furthermore, this last is the algorithm that BCI2000 software, and thus the UMA-BCI Speller, has implemented.

According to the specifications described in the Wiki page of BCI2000^1^, the EEG channels used and their respective weights in the classification matrix are dependent of specific parameters of the user. The different ERP components are commonly found in certain brain zones and certain latencies; but when analyzed particularly for each user, the specific channels and latencies may be different from one another (Luck, 2014). These weights are calculated in the calibration task. The time frame considered to train the classifier was from 0 to 800 ms after the onset of a stimulus (target or non-target). Note that the selection of the channels and calculation of the classification weights were automatically done by the classifier that the BCI2000 software has implemented.

### Spelling Paradigms

**Figure 1:**
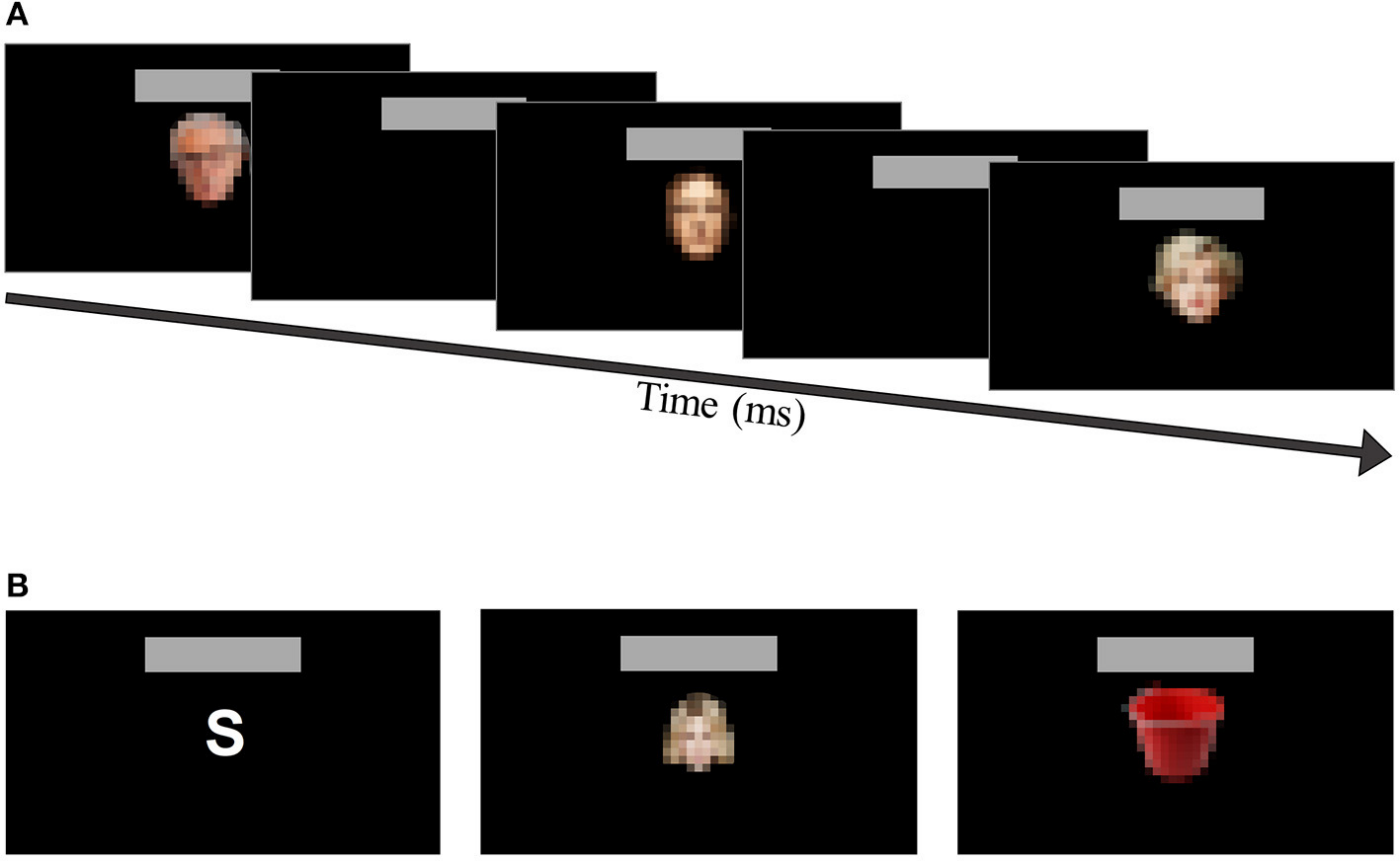
**(A)** RSVP paradigm over time with the Famous Faces (FF) interface as an example; **(B)** Example of a stimulus representation with its equivalent and corresponding white letter (WL, “S”), famous face (FF, “Shakira”) and neutral picture (NP, “*Seau*”). Note that due to copyright reasons, the images presented are pixelated in this figure.

Three different RSVP paradigms were evaluated in the present work. The only difference between paradigms was the type of stimulus used: (i) white letters (WL), (ii) famous faces (FF), and (iii) neutral pictures (NP) (Figure 1).

Each paradigm presented nine different stimuli (Table 1). In the WL paradigm, the letters used were A, B, C, E, L, M, O, R, and S. On the other hand, each character in the FF stimuli was chosen so that the character's name or surname had to start with the same letter as the one used in the WL paradigm (e.g., W. Allen for the letter A, or Beyoncé for the letter B). Finally, for the NP stimuli, the criterion was the same: the picture had to start, in French, with the same letter as the one used in the WL paradigm (e.g., the picture of a tree –*arbre*, in French– for the letter A, or a boat –*bateau*, in French– for the letter B). The relationship between each stimulus and image (face or picture) was explicitly declared by the research staff to participants in order to avoid any mistake. See Table 1 for the letters and their corresponding image names (face and picture). The images used in the experiment are not shown in this paper due to copyright reasons.

**Table 1:** Presentation of the nine stimuli names contained in each set of stimuli.

**Stimuli set**		
**WL**	**FF**	**NP**
A	W. Allen	*Arbre* (tree)
B	Beyoncé	*Bateau* (boat)
C	H. Clinton	*Cloche* (bell)
E	A. Einstein	*Eau* (water)
L	J. Lennon	*Lit* (bed)
M	M. Monroe	*Main* (hand)
O	B. Obama	*Ours* (bear)
R	D. Radcliffe	*Roue* (wheel)
S	Shakira	*Seau* (bucket)

*WL, white letter; FF, famous face; NP, neutral image. Each row contains the letter, famous name, and picture names that are related. The English translation of each word contained in the NP column is presented inside brackets*.

The number of elements was selected in order to avoid a target selection time that was too long, as the aim of this study was to validate the different sets of stimuli under RSVP for communication purposes. In previous studies with this kind of paradigm, an even smaller number of elements has been used to validate hypotheses (Chen et al., 2016; Fernández-Rodríguez et al., 2019c).

The duration of each stimulus presentation was equal to 187.5 ms and the inter stimuli interval (ISI) was equal to 93.75 ms. Therefore, the stimulus onset asynchrony (SOA) had a duration equal to 281.25 ms. The time for completing a sequence (i.e., single presentation or flashing of every stimuli) was 2.44 s. The pause time between one selection and the start of the next (i.e., between completed sets of sequences) was equal to 5 s.

The flashing stimuli were presented in the center of the screen. The dimensions regarding the type of stimuli were as follows: letters, around 3 × 4 cm; faces, around 6 × 8.5 cm; and Pictures, around 12 × 8.5 cm.

### Procedure

A within-subject design was used, so that all users went through all experimental conditions. The experiment was carried out in one session. The order of the paradigms was counterbalanced across participants in order to prevent any undesired effects, such as learning or fatigue. Each condition consisted of two parts: (i) an initial calibration task to obtain the specific signal patterns associated with each user and (ii) an online task in which the user actually controlled the interface. Therefore, the main difference between both tasks was that in the first task the user did not receive any feedback.

For both phases, the task was to write different four-letter words. In the case of the calibration phase, the participant had to write two French words (“MARE,” pond in English, and “CLOS,” enclosed plot in English), so the total number of selections for this task was 8 letters. On the other hand, for the online phase, the user had to write three French words (“MALE,” male in English, “ROSE,” pink in English, and “BOLS,” bowl in English), so the number of selections would be 12 letters. Participants were told during the pause between selections which image (famous face or neutral picture) or letter they have to focus on in the next run. They were not asked to memorize the sets of stimuli used in the experiment (letter, face and picture related) as the purpose of this study was to test the effect of this type of stimuli in a preliminary RSVP-based speller. A short break between words (variable at the request of the user) was employed. The number of sequences (i.e., the number of times that each stimulus –target and non-target– was presented) was pre-fixed to 6 in the calibration task and adapted in the online phase depending on the user performance in the calibration phase. The number of sequences selected for the online task was two trials more than the minimum number of trials required to obtain 100% accuracy in the calibration phase.

At the end of the session, the user had to complete a questionnaire regarding his/her experience during the control of the paradigm.

### Evaluation

Four parameters were used to evaluate the effect of the RSVP paradigm and stimulus type on the performance: (i) the *accuracy* in the calibration and online phases, (ii) the *information transfer rate* (*ITR*) (Wolpaw et al., 1998) in the calibration and online phases, (iii) the analysis of the event-related waveform during the calibration phase, and (iv) a subjective questionnaire.

*Accuracy* was defined as the number of correctly predicted selections divided by the total number of predicted selections, multiplied by 100. While for the online task this last definition was applied, for the calibration phase, the accuracy percentage was computed by the signal classifier after the classification of the word using the data from each sequence. The SWLDA classification algorithm applied was the one proposed by BCI2000.

The *ITR* (bits/min) is an objective measure to determine the communication speed of the system. This parameter considers accuracy, the number of elements available in the interface and time to select one element:

$$ITR=\frac{{log}_{2}\;N\;+\;P\;{log}_{2}\;P\;+\;\left(1\;-\;P\right)\;{log}_{2}\;\frac{1\;-\;P}{N\;-\;1}}{T}\;$$

where *P* is the accuracy of the system, *N* is the number of elements available at the interface and *T* is the time needed to complete a trial (i.e., select an element).

The *ITR* was calculated similarly to the accuracy for both the calibration and the online tasks. It should be noted that the pause between selections was not considered when calculating the *ITR*.

The grand average of the ERP waveforms (from 0 to 800 ms) was analyzed in order to evaluate how the three different stimuli types affected the waveforms of the target, non-target and amplitude difference between target and non-target stimuli. In addition, to carry out a more exhaustive analysis concerning the ERP components frequently used in a BCI, a grand average topography was also carried out for target, non-target and amplitude difference between target and non-target stimuli. Next components were included in the topographical analyses: P100 (60–110 ms), N170 (110–180 ms), P300 (450–520 ms), and N400 (520–570 ms). These topographical maps were statistically compared between conditions. The interval time for each component were chosen according to previous literature and the specific EEG signal obtained in the present study (e.g., Tanaka, 2018; Mijani et al., 2019), except for the N400 component which was selected only according the EEG signal obtained. This last issue is discussed in the Discussion section.

To perform these analyses (i.e., comparison between conditions regarding the grand average of ERP waveforms and the grand average of topography), a baseline from −200 to 0 ms was used for the electrodes, and this was low-pass filtered at 30 Hz. Statistical analyses were carried out using EEGLAB (Delorme and Makeig, 2004), with which a false discovery rate (FDR) correction was applied.

Finally, a subjective questionnaire –specially configured for this experiment– was applied to investigate the experience of the users during the control of the spellers. This questionnaire required that the users scored the different conditions from 0 to 10 using a visual analog scale (VAS) according to the following dimensions: level of fatigue (*fatigue*), complexity of use (*complex*), level of speed felt during presentation of the stimuli (*speed*) and level of stress (*stress*). Where 0 is the lowest value and 10 is the highest for the *fatigue, complex* and *stress* dimensions. For the case of the *speed* dimension, 0 would mean that the interface had an adequate speed of stimuli presentation, and 10 would mean that the speed of the stimuli presentation was too fast.

## Results

In this section, the different results are presented in different sections. First, the results of the calibration task (i.e., performance metrics and ERP waveforms) are presented, followed by the performance metrics of the online phase, and finally, the subjective questionnaire analysis.

### Calibration Task

#### Performance Metrics

**Figure 2:**
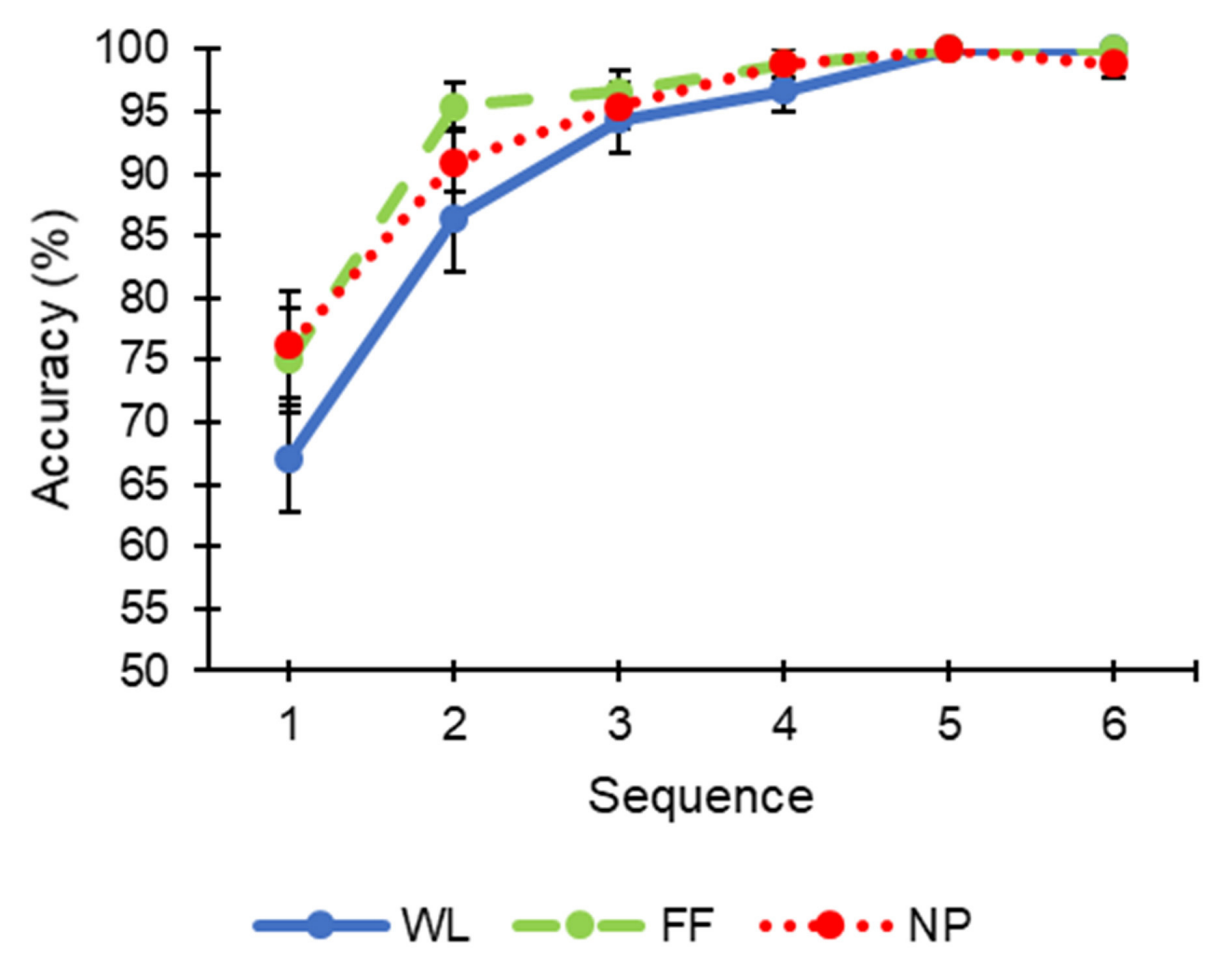
Accuracy (mean ± standard error) of each condition (WL, white letters; FF, familiar faces; NP, neutral pictures) as a function of the number of sequences during the calibration task.

**Figure 3:**
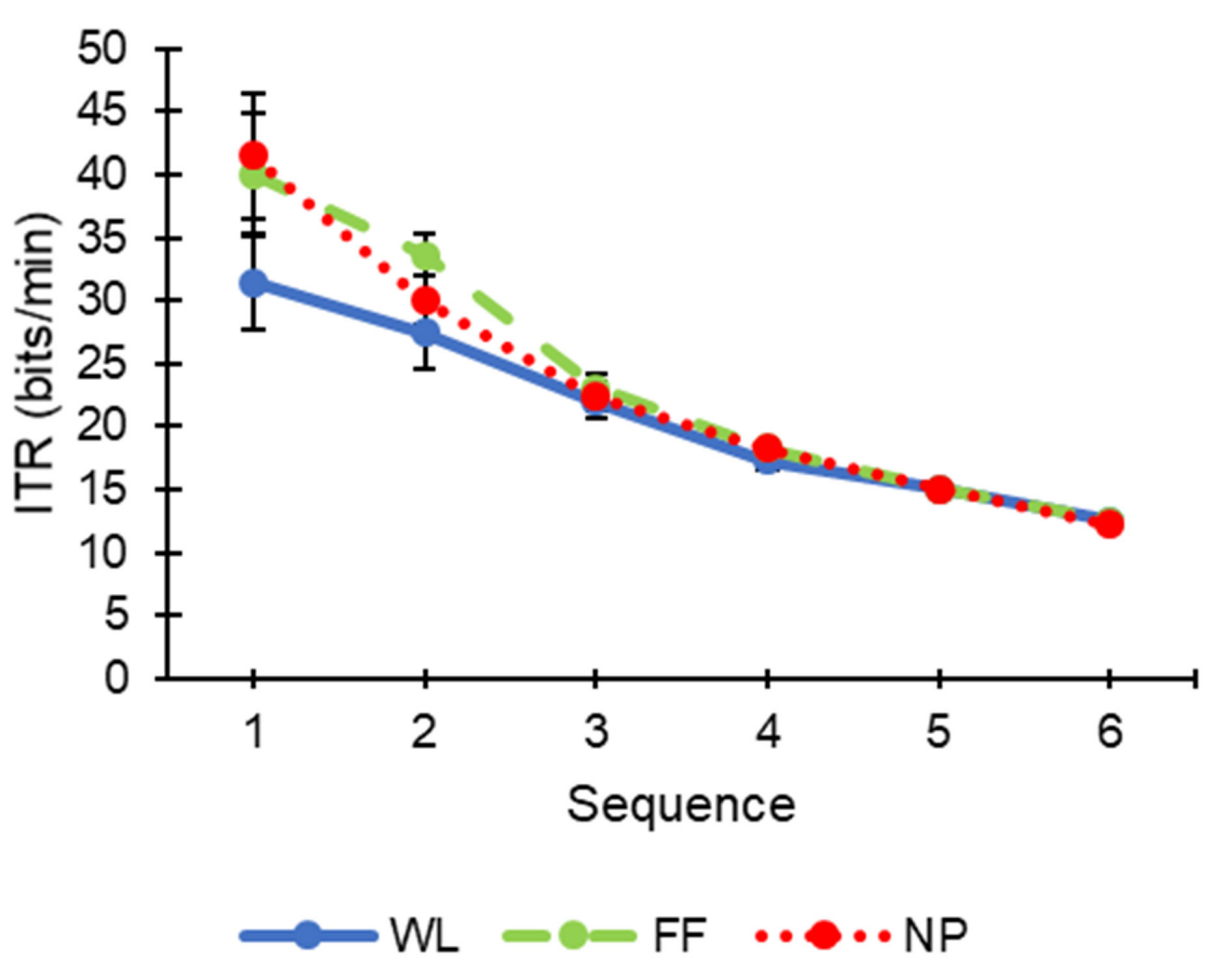
Information transfer rate (ITR, mean ± standard error) of each condition (WL, white letters; FF, familiar faces; NP, neutral pictures) as a function of the number of sequences during the calibration task.

In order to find out if there were any significant differences between the different conditions, a Student's *t*-test was performed for repeated samples for each of the sequences. The *accuracy* (Figure 2) did not show significant differences for any sequence. However, the variable *ITR* (Figure 3) showed significant differences for the first sequence between conditions WL and NP [*t*_(10)_ = 2.24; *p* = 0.049] (Supplementary Table 1). Likewise, some marginally significant differences were revealed when the average *accuracy* and *ITR* of all sequences were calculated (WL, 90.74 ± 5.44% and 20.95 ± 3.88 bits/min; FF, 94.32 ± 3.5% and 23.75 ± 3.34 bits/min; NP, 93.39 ± 4.36% and 23.23 ± 4 bits/min). Specifically, WL was observed to offer a marginally significant worst performance than FF [*accuracy, t*_(10)_ = 2.161; *p* = 0.056; *ITR, t*_(10)_ = 2.175; *p* = 0.055] and NP [*ITR, t*_(10)_ = 1.89; *p* = 0.088].

#### ERP Waveform

**Figure 4:**
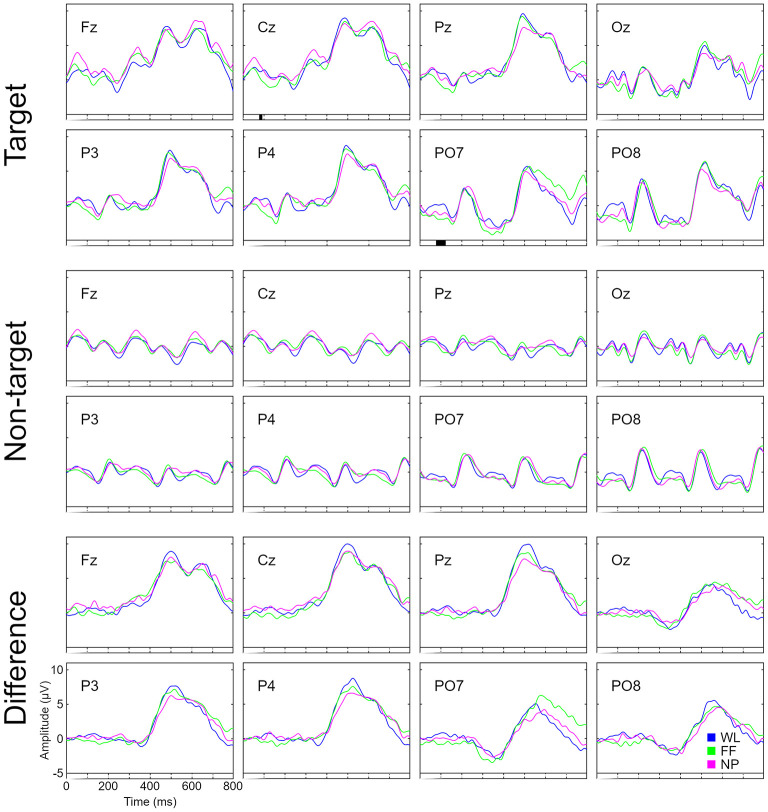
Grand average ERP waveform for target, non-target and amplitude difference between target and non-target stimuli signals in all used channels (Fz, Cz, Pz, Oz, P3, P4, PO7, and PO8) for the three conditions: white letters (WL), familiar faces (FF), and neutral pictures (NP). These plots were obtained from the EEG data recorded during the calibration phase.

Regarding the grand average ERP waveform, the statistical analyses showed significant differences between conditions at an early time interval (around 80–110 ms) for target stimuli in Cz and PO7 (Figure 4).

**Figure 5:**
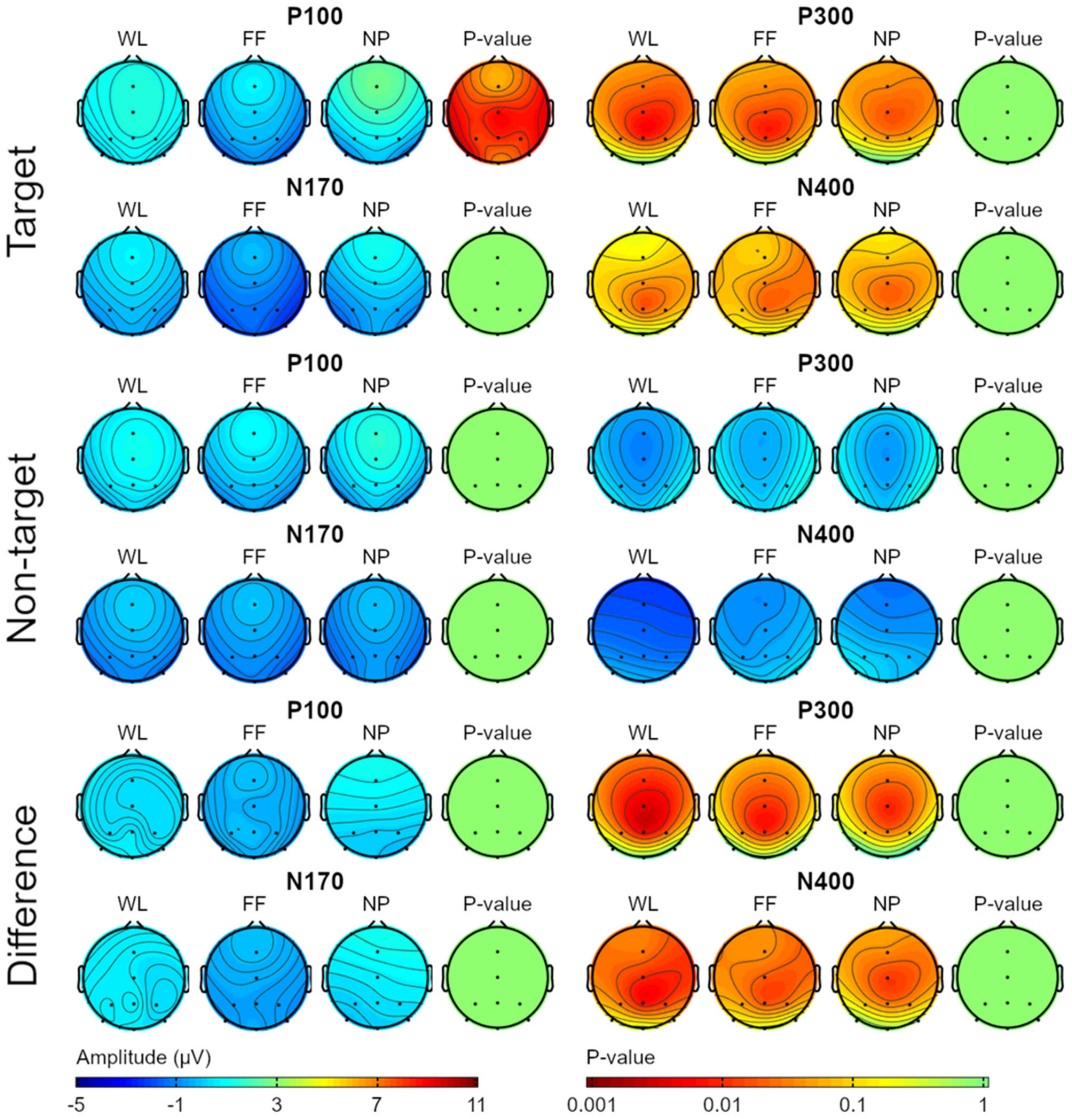
Topographical scalp map of each condition (WL, white letters; FF, familiar faces; NP, neutral pictures) for the next components: P100 (60–110 ms), N170 (110–180 ms), P300 (450–520 ms), and N400 (520–570 ms). These plots were obtained from the EEG data recorded during the calibration phase.

On the other hand, regarding the grand average topography of the P100, N170, P300, and N400 components, only the P100 (60–110 ms) component showed significant differences in channels Cz, Pz, Oz, P3, P4, PO7, and PO8 for the target stimuli (Figure 5). These differences could indicate a difference in early processing depending on the type of visual stimulus. Specifically, it appeared that the FF condition obtained lower grand average ERP amplitude values than those obtained by the WL and NP conditions (Figure 4).

### Online Task

The *accuracy* and *ITR* results achieved, as well as the number of sequences used by each participant in the online task, are shown in Table 2. In regard to the *accuracy* obtained for the online task (second main column of Table 2), the Student's *t-*test between conditions showed no significant differences between NP and the rest of conditions [NP vs. WL, *t*_(10)_ = 0.183; *p* = 0.859; NP vs. FF, *t*_(10)_ = 0.957; *p* = 0.361]. However, a comparison between WL and FF conditions showed a trend close to significance [*t*_(10)_ = 1.961; *p* = 0.078]. On the other hand, for the *ITR* (third main column of Table 2), significant differences were found between the WL and FF conditions [*t*_(10)_ = 2.973; *p* = 0.014], but not between WL and NP [*t*_(10)_ = 0.595; *p* = 0.565] nor NP and FF [*t*_(10)_ = 1.261; *p* = 0.236].

**Table 2:** Accuracy (%), ITR (bits/min) and number of sequences used (mean ± standard deviation) of the three conditions (WL, white letters; FF, familiar faces; NP, neutral pictures) during the online task.

**User**	**Accuracy**			**ITR**			**Number of sequences**		
	**WL**	**FF**	**NP**	**WL**	**FF**	**NP**	**WL**	**FF**	**NP**
U1	79.2	87.5	100	14.29	26.68	18.78	3	2	4
U2	83.3	100	66.7	9.57	15.03	5.94	5	5	5
U3	79.2	95.8	87.5	14.29	16.55	17.79	3	4	3
U4	95.8	100	95.8	22.06	25.04	33.10	3	3	2
U5	91.7	91.7	87.5	9.91	14.86	17.79	6	4	3
U6	87.5	83.3	95.8	8.89	15.95	13.24	6	3	5
U7	87.5	75	87.5	13.34	12.71	17.79	4	3	3
U8	91.7	100	83.4	19.82	25.04	7.99	3	3	6
U9	75	83.3	62.5	7.63	7.97	6.46	5	6	4
U10	100	100	87.5	18.78	15.03	10.67	4	5	5
U11	66.7	79.2	91.67	7.42	14.29	9.90	4	3	6
Mean	85.24 ± 9.74	90.53 ± 9.33	85.99 ± 11.67	13.27 ± 5.13	17.2 ± 5.86	14.5 ± 7.84	4.18 ± 1.17	3.73 ± 1.19	4.18 ± 1.33

### Subjective Questionnaires

**Figure 6:**
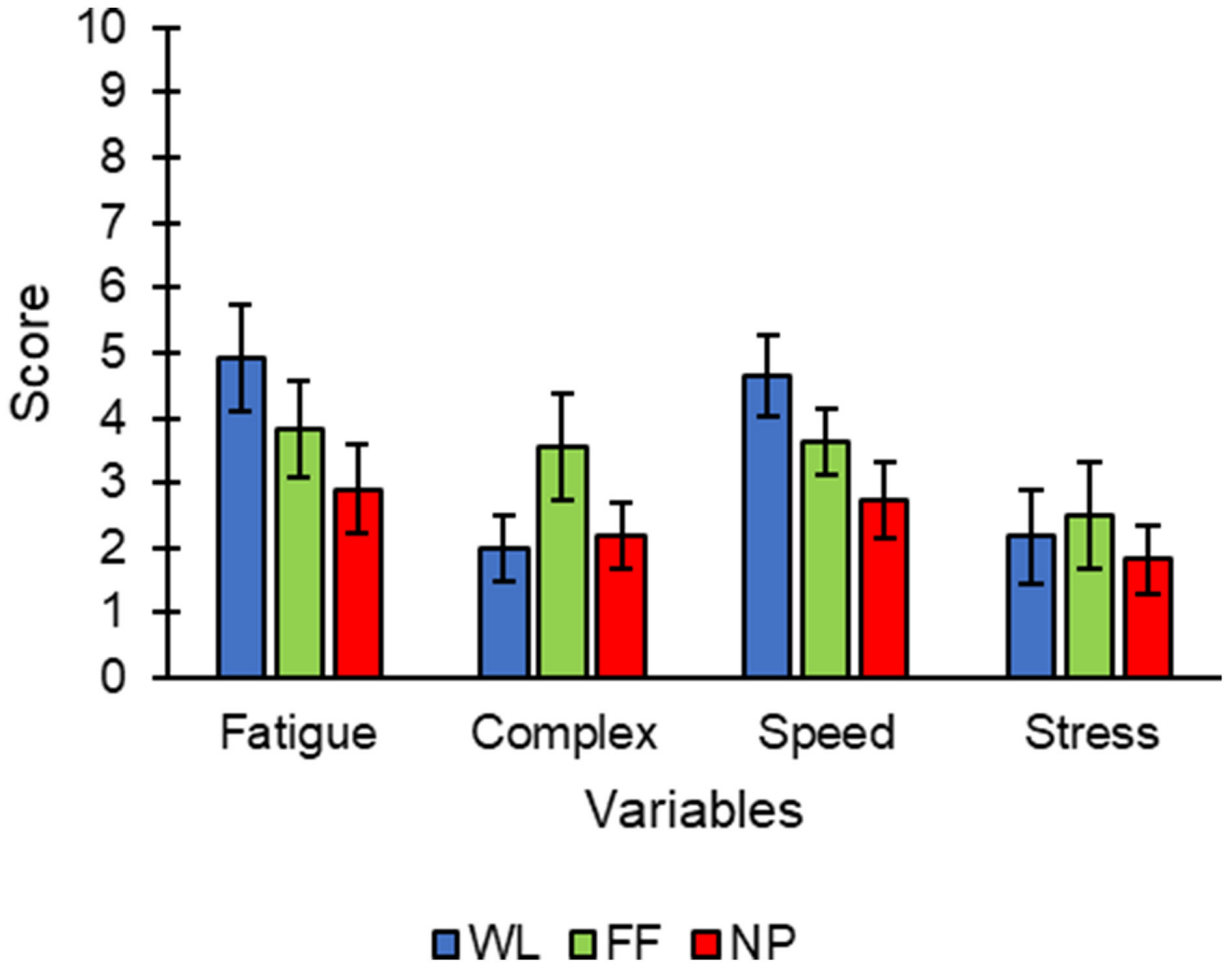
Scores (mean ± standard error) of each condition (WL, white letters; FF, familiar faces; NP, neutral pictures) for the variables collected in the subjective questionnaire.

With reference to the results obtained in the questionnaire (Figure 6), the condition NP, compared to WL, was found to be associated with significantly less *fatigue* [*t*_(10)_ = 2.262; *p* = 0.047] and more appropriate speed presentation –*speed*– [*t*_(10)_ = 3.13; *p* = 0.011]. Note that the comparison of FF and NP was near nominal significance for *speed* [*t*_(10)_ = 2.085; *p* = 0.064]. All statistical comparisons made between conditions for the subjective questionnaires can be observed in Supplementary Table 2.

## Discussion

In this study we tested different kinds of stimuli –white letters (WL), famous faces (FF) and neutral picture (NP)– under a rapid serial visual presentation (RSVP) paradigm to analyse the system performance in terms of classification accuracy, information transfer rate (ITR), ERP waveform and user experience (*fatigue, complexity, speed*, and *stress* level). Main results showed that FF and NP might produce, respectively, better performance and better user experience compared to WL. These results suggest that the stimuli proposed (FF and NP) could enhance the system performance, and thus communication, of this type of gaze-independent BCI.

### Calibration Task

#### Performance Metrics

Main results regarding *accuracy* showed that, in the first sequence, the NP condition had a significantly higher *ITR* in contrast to the WL condition. These results are especially interesting for those cases in which higher communication speed is preferred even though *accuracy* is partially decreased. In fact, the *accuracy* reached by the NP condition in the first sequence (76.18 ± 14.24%) was higher than 70%, which is the minimum *accuracy* recommended by Kübler et al. (2001), and normally used by the BCI community, to enable an efficient communication system. The FF condition achieved similar results in *accuracy* and *ITR* (75 ± 13.69% and 40.01 ± 16.1 bits/min) to the NP (76.18 ± 14.24% and 41.46 ± 16.62 bits/min) in the first sequence, but it was still slightly lower and, thus, did not reach statistical significance when compared with WL, neither for *accuracy* nor *ITR* (WL: 67.01 ± 13.99% and 31.45 ± 12.58 bits/min).

For the rest of sequences, it can be observed that the higher the number of sequences the more similar the results for the different conditions (Figures 2, 3). Nevertheless, from the average *accuracies* and *ITR*s throughout the sequence, it was observed that the values of the FF and NP conditions showed marginally significant better performance than the WL condition (*accuracy, p* = 0.056; *ITR, p* = 0.055). Therefore, the tendency is toward the WL condition showing a worse performance than FF and NP.

#### ERP Waveform

Significant differences were obtained in the analysis of the grand average ERP waveform, particularly in early time intervals in channels Cz and PO7 for target stimuli between conditions (Figure 4). These significances were corroborated in the topographical analyses (Figure 5). The component P100 (60–110 ms) offered significant differences between conditions. Thus, it can be affirmed that there are differences in early neural processing depending on the type of visual stimulus. Specifically, it appeared that the FF condition obtained lower grand average ERP amplitude values than those obtained by the WL and NP conditions. Furthermore, observing the grand average ERP amplitude at the following milliseconds (Figure 4), a possible N170 component is observed in the three conditions in almost every channel. Nevertheless, this potential is especially pronounced for the FF condition –although not significant– in contrast to those obtained by the WL and NP conditions. These results would fit with previous BCI literature, as N170 is a potential related to facial recognition (Kaufmann et al., 2011; Kellicut-Jones and Sellers, 2018).

Regarding later potentials, the P300 potential was clearly the most distinctive and largest component in all the channels for the three conditions. On the other hand, the N400 –which is related to familiar face recognition– was not found as reported in previous studies (Dijkstra et al., 2020). We deduced that it might have different latency because of the stimuli presentation used, or that it might have been delayed or even partially canceled by the P300 component (which had large –but common– amplitude and latency). Most probably, the N400 potential was not found in the present study as the paradigm applied in this experiment did not use any type of semantic incongruity, which have been related by the literature with the increase in the N400 potential (Eimer, 2000).

The function of the classifier is to discriminate between target and non-target stimuli. The positive correlation between amplitude of ERP waveform and performance in a visual ERP-based BCI has been previously demonstrated (Mak et al., 2012). It could be considered that a larger difference between target and non-target stimuli for any of the studied ERP components could increase the classifier performance. Thus, the results obtained in the ERP waveforms (Figures 4, 5) might correlate with what was obtained in the calibration phase regarding performance (Figures 2, 3 and Supplementary Table 1). Specifically, the significant differences obtained in the first sequence of the ITR variable (Figure 3), between WL and NP, could be related to those found in the P100 component (Figure 5). Likewise, the higher performance of FF vs. WL in the first sequences of the calibration phase could be related to the grand average ERP amplitude of the N170 component presented for FF (Figure 4).

### Online Task

For the online task, the FF condition achieved a significantly higher *ITR* as compared to the WL condition (17.2 ± 5.86 and 13.27 ± 5.13 bits/min, respectively), and a higher *accuracy*, which showed a trend close to significance (*p* = 0.078), was observed between these two conditions (WL, 85.24 ± 9.74%; FF, 90.53 ± 9.33%). On the other hand, the performance of the NP condition (85.99 ± 3.52%, 14.5 ± 2.36 bits/min) seemed to be placed in the middle and no significant differences were revealed as compared to the other two conditions (Table 2). Therefore, once again, the WL condition was found to be the least appropriate for the RSVP paradigm. These found observations go in the same direction as other authors suggest that the WL condition could be the less appropriate for the RCP paradigm than FF (Kaufmann et al., 2011; Kellicut-Jones and Sellers, 2018).

Comparing the obtained results with those of previous studies that also assessed RSVP spellers using only letters as stimuli, it can be observed that the reported *accuracy* and *ITR* values of this study are similar to those reported in the literature (Acqualagna and Blankertz, 2013; Chennu et al., 2013; Lin et al., 2018; Won et al., 2018; Fernández-Rodríguez et al., 2019c). To the best of our knowledge, the FF condition has not been used before for communication purposes under RSVP, and the NP condition has only been evaluated in a preliminary study (Fernández-Rodríguez et al., 2019c). Thus, the performance achieved by the FF or NP paradigms cannot be fairly compared to any other study, highlighting the novelty of this work.

The performance of the present study (especially the ITR) can be consider lower than the performance obtained by those studies that applied the RCP paradigm. This lower performance is essentially related to the time needed by the RSVP paradigm to present every stimuli in comparison to the one needed by RCP (Chennu et al., 2013). However, it should be noted that the RCP paradigm needs ocular mobility to be efficiently controlled what might limit its use for some patients (Brunner et al., 2010; Treder and Blankertz, 2010).

### Subjective Questionnaire

Remarkably, the overall results of the subjective questionnaire were positive for the three interfaces, since all the average values were below 5 points (considering that the highest possible score was 10) for different subjective measurements (*fatigue, complex, speed*, or *stress*): WL, between 2 and 5 points (3.43 ± 2.56); FF, between 3 and 4 points (3.38 ± 2.35); and NP between 1 and 3 points (2.41 ± 1.87). Regarding the specific variables, the NP condition seemed to be the condition that gave the best results in terms of fatigue produced (*fatigue*) and interface speed adequacy (*speed*). In fact, NP offered a lower *fatigue* and *speed* vs. WL. Also, it is worth noting that the NP condition was near nominal significant to show better results than FF in the *speed* variable (*p* = 0.064). In addition to *fatigue* and *speed*, the NP condition showed the best results (i.e., the lowest values) for the *stress* parameter. On the other hand, the FF condition showed the highest scores for *complex* (although this was not significant). This last result is in contrast with observations at a global level for both the calibration and the online task, where FF generally presented better results.

Interestingly, the ERP waveforms (Figures 4, 5) might correlate with what was declared by the participants regarding their subjective perspective of the paradigms (Figure 6 and Supplementary Table 2). First, most probably we could not find more statistically differences in the ERP waveforms because the overall results of the subjective questionnaire were positive for the three spellers. Therefore, even though the NP condition obtained the best results for the *fatigue, speed* and *stress* parameters, these improvements might not highly affect the brain signal. Furthermore, the FF paradigm was declared as the most *complex* in a non-significant manner. This could be related to the non-significant differences obtained in the P300 potential, an ERP component previously associated with the complexity of the task (Käthner et al., 2014).

These results should be considered, especially in those cases where these applications want to be controlled during long sessions (either in the case of patients or healthy users), in which high levels of fatigue can diminish both user performance and satisfaction (Käthner et al., 2014).

### Future Studies

Future studies might investigate more deeply why the effect of pictures or faces has not been as great as that observed for RCP in previous works (e.g., Kaufmann et al., 2011 and Fernández-Rodríguez et al., 2019a). Likewise, it would be interesting to further study whether the novel findings obtained under RCP in reference to the applied stimuli –for example, green famous faces, self-face paradigm or very small lateral stimuli (Li et al., 2015; Xu et al., 2018; Lu et al., 2020, respectively)– can be transferred to RSVP.

Furthermore, there are different BCI works in the literature that propose paradigms with reduced number of stimuli such as target selection in consecutive steps (Treder et al., 2011) or the T9 keyboard (Ron-Angevin et al., 2015). It would be interesting to test our proposed stimuli (face or picture) in this sort of reduced paradigms.

Finally, a further research to improve the system performance of the presented paradigms with images (face and pictures) would be also interesting. These improvements could be related to the type of classification algorithm used (Xiao et al., 2020), the creation of a generic model to decrease the calibration time (Jin et al., 2020), or even the application of hybrid systems which use different type of control signals (Xu et al., 2020).

## Conclusion

The aim of this work was to assess the impact of three different types of stimuli under RSVP for communication purposes: WL, FF, and NP. In general terms, it seems that both the FF and NP conditions have a tendency to offer a better performance as compared to the WL condition, either for objective measurements (both for FF and NP in the calibration, and for FF in the online task) or for subjective measurements (in particular for NP).

Concerning any comparison between FF and NP, it is difficult to choose a recommended approach for potential users, because while the online task proved better for the FF condition, the NP condition achieved better scores in the subjective questionnaires. It is worth considering whether this performance improvement is more important than considering the subjective preference of the NP interface. It should be remembered that there were no significant differences between FF and NP throughout the study. Therefore, we estimate that the choice between the use of FF or NP will depend on the specific conditions and preferences of each user. However, it is clear that the WL condition should seldom be considered as the most suitable choice for a user.

## Data Availability Statement

The raw data supporting the conclusions of this article will be made available by the authors, without undue reservation.

## Ethics Statement

The studies involving human participants were reviewed and approved by Comité Ético de Experimentación de la Universidad de Málaga (CEUMA). CEUMA registry number: 51-2019-H. The patients/participants provided their written informed consent to participate in this study.

## Author Contributions

RR-A, LG, MM-J, J-MA, and VL-N contributed to the conception and design of the study. ÁF-R, MM-J, and FV-Á performed the statistical analysis. MM-J and ÁF-R wrote the first draft of the manuscript. RR-A and LG were in charge of the supervision of the project. All authors contributed to manuscript revision, read, and approved the submitted version.

## Conflict of Interest

The authors declare that the research was conducted in the absence of any commercial or financial relationships that could be construed as a potential conflict of interest.

## Footnotes

^1^https://www.bci2000.org/mediawiki/index.php/Main_Page
