# Supplementary material for: Performance Analysis With Different Types of Visual Stimuli in a BCI-Based Speller Under an RSVP Paradigm
Source: Front Comput Neurosci. 2021 Jan 5;14:587702. doi: 10.3389/fncom.2020.587702 (PMC7814000; doi:10.3389/fncom.2020.587702)
Supplement: Supplementary file 1 [file Table_1.docx]

Supplementary Material

# Statistical analysis results from the calibration task

| **Table S1 \|** Accuracy and information transfer rate (ITR) comparisons between conditions for each sequence in the calibration task. | | | | |
| --- | --- | --- | --- | --- |
| **Sequence** | **Variables** | **Comparison** | | |
|  |  | **WL-FF** | **WL-NP** | **FF-NP** |
| Sequence 1 | Accuracy | *t* (10) = 1.34; *p* = 0.21 | *t* (10) = 2.025; *p* = 0.07 | *t* (10) = 0.228; *p* = 0.825 |
|  | ITR | *t* (10) = 1.424; *p* = 0.185 | ***t* (10) = 2.24; *p* = 0.049** | *t* (10) = 0.22; *p* = 0.831 |
| Sequence 2 | Accuracy | *t* (10) = 1.789; *p* = 0.104 | *t* (10) = 1.077; *p* = 0.307 | *t* (10) = 1.481; *p* = 0.169 |
|  | ITR | *t* (10) = 1.742; *p* = 0.112 | *t* (10) = 0.877; *p* = 0.401 | *t* (10) = 1.404; *p* = 0.191 |
| Sequence 3 | Accuracy | *t* (10) = 0.803; *p* = 0.441 | *t* (10) = 0.363; *p* = 0.724 | *t* (10) = 0.559; *p* = 0.588 |
|  | ITR | *t* (10) = 0.753; *p* = 0.469 | *t* (10) = 0.276; *p* = 0.788 | *t* (10) = 0.559; *p* = 0.588 |
| Sequence 4 | Accuracy | *t* (10) = 0.989; *p* = 0.346 | *t* (10) = 0.989; *p* = 0.346 | *t* (10) = 0; *p* = 1 |
|  | ITR | *t* (10) = 0.991; *p* = 0.345 | *t* (10) = 0.991; *p* = 0.345 | *t* (10) = 0; *p* = 1 |
| Sequence 5 | Accuracy | not calculated ^a^ | not calculated ^a^ | not calculated ^a^ |
|  | ITR | not calculated ^a^ | not calculated ^a^ | not calculated ^a^ |
| Sequence 6 | Accuracy | not calculated ^a^ | *t* (10) = 1; *p* = 0.341 | *t* (10) = 1; *p* = 0.341 |
|  | ITR | not calculated ^a^ | *t* (10) = 1; *p* = 0.341 | *t* (10) = 1; *p* = 0.341 |
| Average | Accuracy | *t* (10) = 2.161; *p* = 0.056 | *t* (10) = 1.57; *p* = 0.148 | *t* (10) = 0.686; *p* = 0.509 |
|  | ITR | *t* (10) = 2.175; *p* = 0.055 | *t* (10) = 1.89; *p* = 0.088 | *t* (10) = 0.374; *p* = 0.716 |
| *^a^ The t-value cannot be calculated because the standard error of the differences is equal to zero (both compared conditions obtained the same accuracy for each subject).  Significant differences are highlighted in bold.* | | | | |

# Statistical analysis results from the subjective questionnaire

| **Table S2 \|** Subjective questionnaire’s comparisons between conditions. | | | |
| --- | --- | --- | --- |
| **Variables** | **Comparison** | | |
|  | **WL-FF** | **WL-NP** | **FF-NP** |
| Fatigue | *t* (10) = 0.865; *p* = 0.407 | ***t* (10) = 2.262; *p* = 0.047** | *t* (10) = 0.922; *p* = 0.378 |
| Complex | *t* (10) = 1.599; *p* = 0.141 | *t* (10) = 0.377; *p* = 0.714 | *t* (10) = 1.487; *p* = 0.168 |
| Speed | *t* (10) = 1.454; *p* = 0.176 | ***t* (10) = 3.13; *p* = 0.011** | *t* (10) = 2.085; *p* = 0.064 |
| Stress | *t* (9) = 0.269; *p* = 0.794 | *t* (10) = 1.174; *p* = 0.267 | *t* (9) = 1.068; *p* = 0.313 |
| *The significant differences found in this analysis are highlighted in bold. For unknown reasons, participant 3 did not answer the item regarding the stress for FF condition.* | | | |
